# IP_3_R2 levels dictate the apoptotic sensitivity of diffuse large B-cell lymphoma cells to an IP_3_R-derived peptide targeting the BH4 domain of Bcl-2

**DOI:** 10.1038/cddis.2013.140

**Published:** 2013-05-16

**Authors:** H Akl, G Monaco, R La Rovere, K Welkenhuyzen, S Kiviluoto, T Vervliet, J Molgó, C W Distelhorst, L Missiaen, K Mikoshiba, J B Parys, H De Smedt, G Bultynck

**Affiliations:** 1 Laboratory of Molecular and Cellular Signaling, Department of Cellular and Molecular Medicine, KU Leuven, Leuven, Belgium; 2 Laboratory of Physiology, Department of Neuroscience and Imaging, University of G. d'Annunzio, Chieti, Italy; 3 CNRS, Institut Fédératif de Neurobiologie Alfred Fessard, Laboratoire de Neurobiologie et Développement UPR3294, Gif-sur-Yvette, France; 4 Department of Medicine, Case Western Reserve University and University Hospitals of Cleveland, Cleveland, Ohio, USA; 5 Laboratory for Developmental Neurobiology, RIKEN Brain Science Institute, Wako, Japan

**Keywords:** Ca^2+^ signaling, Bcl-2, IP_3_ receptors, cell death, apoptosis, B-cell lymphoma

## Abstract

Disrupting inositol 1,4,5-trisphosphate (IP_3_) receptor (IP_3_R)/B-cell lymphoma 2 (Bcl-2) complexes using a cell-permeable peptide (stabilized TAT-fused IP_3_R-derived peptide (TAT-IDP^S^)) that selectively targets the BH4 domain of Bcl-2 but not that of B-cell lymphoma 2-extra large (Bcl-Xl) potentiated pro-apoptotic Ca^2+^ signaling in chronic lymphocytic leukemia cells. However, the molecular mechanisms rendering cancer cells but not normal cells particularly sensitive to disrupting IP_3_R/Bcl-2 complexes are poorly understood. Therefore, we studied the effect of TAT-IDP^S^ in a more heterogeneous Bcl-2-dependent cancer model using a set of ‘primed to death' diffuse large B-cell lymphoma (DL-BCL) cell lines containing elevated Bcl-2 levels. We discovered a large heterogeneity in the apoptotic responses of these cells to TAT-IDP^S^ with SU-DHL-4 being most sensitive and OCI-LY-1 being most resistant. This sensitivity strongly correlated with the ability of TAT-IDP^S^ to promote IP_3_R-mediated Ca^2+^ release. Although total IP_3_R-expression levels were very similar among SU-DHL-4 and OCI-LY-1, we discovered that the IP_3_R2-protein level was the highest for SU-DHL-4 and the lowest for OCI-LY-1. Strikingly, TAT-IDP^S^-induced Ca^2+^ rise and apoptosis in the different DL-BCL cell lines strongly correlated with their IP_3_R2-protein level, but not with IP_3_R1-, IP_3_R3- or total IP_3_R-expression levels. Inhibiting or knocking down IP_3_R2 activity in SU-DHL-4-reduced TAT-IDP^S^-induced apoptosis, which is compatible with its ability to dissociate Bcl-2 from IP_3_R2 and to promote IP_3_-induced pro-apoptotic Ca^2+^ signaling. Thus, certain chronically activated B-cell lymphoma cells are addicted to high Bcl-2 levels for their survival not only to neutralize pro-apoptotic Bcl-2-family members but also to suppress IP_3_R hyperactivity. In particular, cancer cells expressing high levels of IP_3_R2 are addicted to IP_3_R/Bcl-2 complex formation and disruption of these complexes using peptide tools results in pro-apoptotic Ca^2+^ signaling and cell death.

B-cell lymphoma 2 (Bcl-2) as an anti-apoptotic protein has a central role in regulating cell death and survival.^1^ Altered Bcl-2 biology has been implicated in a large number of cancer cells, including B-cell lymphomas like diffuse large B-cell lymphoma (DL-BCL) and chronic lymphocytic leukemia (CLL).^2, 3, 4^ In many cases, Bcl-2 is upregulated, increasing the resistance of the cancer cell toward pro-apoptotic signals like oncogenic stress or genomic instability and thus promoting their survival.^5, 6^

At the cellular level, the anti-apoptotic function of Bcl-2 is mediated by neutralizing pro-apoptotic Bcl-2-family members, including the executioner proteins Bax and Bak, the activator BH3-only proteins Bid and Bim, and the sensitizer/de-repressor BH3-only proteins Bad and others.^5, 6^ Bcl-2 binds to the BH3 domain of these pro-apoptotic Bcl-2-family members through its hydrophobic cleft formed by the BH1, -2 and -3 domains. In this modus, Bcl-2 targets the mitochondria, preventing apoptosis initiation through cytochrome C release.^7, 8^ Some cancer cells are addicted to high Bcl-2 levels to neutralize upregulated BH3-only proteins like Bim.^6, 9, 10^ These cells are very sensitive to BH3-mimetic drugs like ABT-737, which compete with Bim for binding to Bcl-2, resulting in Bim release from Bcl-2 and apoptosis initiation.^10, 11, 12^

Bcl-2, however, also indirectly protects against mitochondria-mediated apoptosis by targeting the endoplasmic reticulum (ER) Ca^2+^ store.^13, 14, 15^ Although Bcl-2 might lower ER Ca^2+^ levels,^16, 17^ there is now strong evidence that Bcl-2 directly binds and inhibits inositol 1,4,5-trisphosphate (IP_3_) receptors (IP_3_Rs).^18^ Recently, we found that Bcl-2 suppresses IP_3_R activity through its BH4 domain, preventing the pro-apoptotic Ca^2+^ flux from the ER into mitochondria.^19^ We identified the Bcl-2-binding site on the IP_3_R and developed a peptide corresponding to this binding site (indicated here as TAT-IDP for TAT-conjugated IP_3_R-derived peptide). This peptide disrupted Bcl-2 binding and severely enhanced IP_3_R-mediated Ca^2+^ signals with pro-apoptotic properties.^20^ As a result, TAT-IDP-treated T lymphocytes displayed an increased sensitivity toward pro-apoptotic stimuli (like strong T-cell-receptor stimulation). Furthermore, we also developed a stabilized, protease-resistant form of the peptide (TAT-IDP_DD/AA_, which will be further indicated as TAT-IDP^S^). TAT-IDP^S^ provoked pro-apoptotic Ca^2+^ signals in CLL patient cells.^21^ Hence, in contrast to normal cells, which were resistant to TAT-IDP^S^ by itself but displayed enhanced sensitivity toward apoptotic triggers, CLL patient cells underwent apoptotic cell death in the presence of the peptide alone. This raises the question whether cancer cells, in particular Bcl-2-dependent malignancies, displayed altered Ca^2+^-signaling properties that turned these cells into vulnerable targets toward peptides disrupting Bcl-2-mediated suppression of apoptotic IP_3_R activity. Importantly, these peptides selectively target the BH4 domain of Bcl-2, but not that of Bcl-Xl.^22^

Here, we studied a set of cell lines derived from DL-BCL tumors, a disease characterized by its heterogeneity in gene expression, oncogenic aberrations, intrinsic apoptotic escape routes, and response to chemotherapy.^23, 24, 25^ In particular, we focused on BH3-profiled ‘primed to death' DL-BCL cell lines that are dependent on Bcl-2 upregulation.^26^ We found that the relative IP_3_R2-expression level was an important determinant for the apoptotic response of these cells, and correlated with the ability of TAT-IDP^S^ to trigger pro-apoptotic IP_3_R-mediated Ca^2+^ release. We found that disrupting Bcl-2 binding to IP_3_Rs was particularly effective in cancer cells with high levels of IP_3_R2. The presence of IP_3_R2 rendered these cells vulnerable toward ongoing IP_3_ signaling, for example, like during chronic activation, whereas cells expressing relatively low levels of IP_3_R2 were much less sensitive. Such a correlation was not observed for the two other IP_3_R isoforms (IP_3_R1 and IP_3_R3). Hence, this is the first study to reveal a prominent role for the type of IP_3_R isoform as a determinant for the sensitivity to cell death in Bcl-2-dependent cancer cell lines.

## Results

### Some types of ‘primed to death' DL-BCL cells are sensitive to TAT-IDP^S^

**Figure 1:**
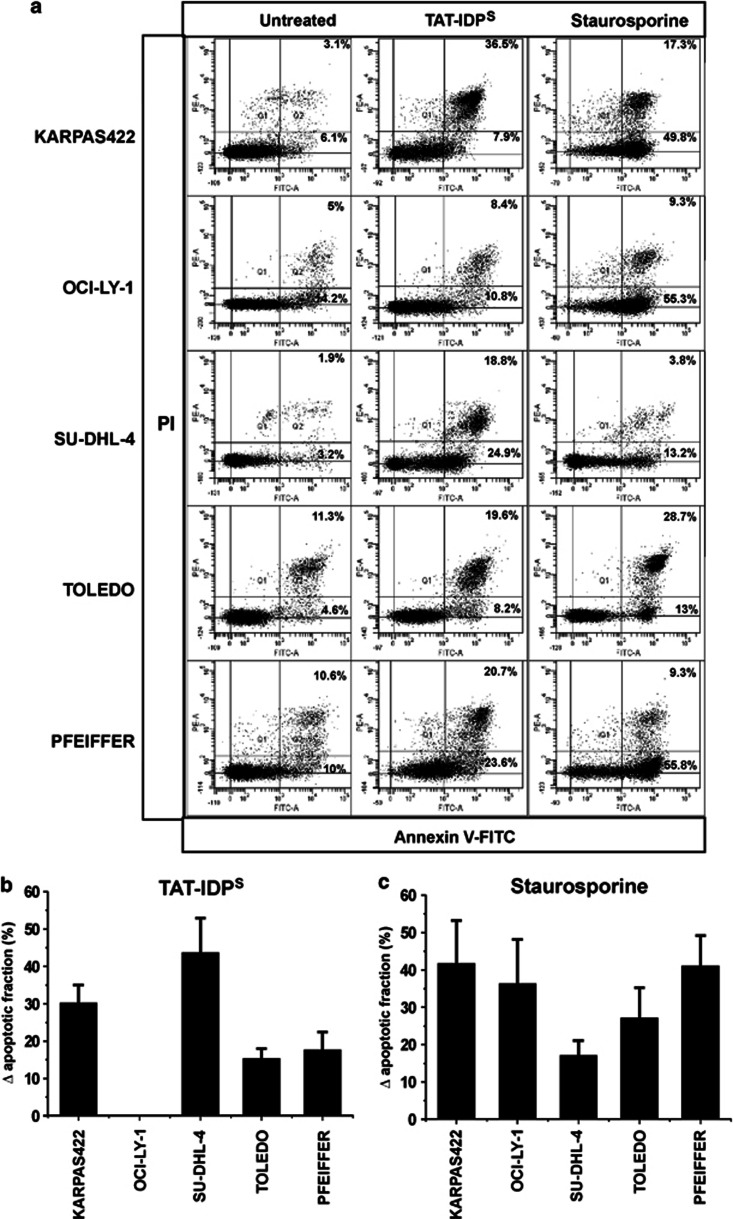
TAT-IDP^S^-induced apoptosis in DL-BCL cell lines. (**a**) Representative dot plots from flow cytometry analysis of apoptosis using annexin V-FITC/PI-stained KARPAS422, OCI-LY-1, SU-DHL-4, TOLEDO, and PFEIFFER, either untreated or treated with 10 *μ*M TAT-IDP^S^ for 24 h or with 1 *μ*M staurosporine for 6 h (10 000 cells per analysis). The apoptotic population was identified as the annexin V-FITC-positive fraction (Q2+Q4). (**b**, **c**) Quantitative analysis of four independent experiments of the (**b**) TAT-IDP^S^- and (**c**) staurosporine-induced apoptosis (Δ apoptotic fraction=apoptotic population in treated cells−apoptotic population in untreated cells). Data are expressed as the average ±S.D.

In a primary screening, we investigated five well-characterized and ‘primed to death' DL-BCL lines, previously BH3 profiled by the laboratory of Dr. A. Letai (KARPAS422, TOLEDO, PFEIFFER, SU-DHL-4, and OCI-LY-1).^6, 12^ KARPAS422, TOLEDO, SU-DHL-4, and OCI-LY-1 display upregulated Bcl-2 levels and high amounts of Bcl-2/Bim complexes, rendering these cells particularly sensitive toward the BH3-mimetic drug ABT-737,^12^ whereas PFEIFFER displayed relatively high levels of Bfl-1 and myeloid-cell leukemia 1 (Mcl-1) levels making this cell line more resistant to ABT-737.^12^ Strikingly, we found remarkable differences in the response of these DL-BCL cells toward TAT-IDP^S^ exposure (10 *μ*M, 24 h) in cell-death experiments based on annexin V-FITC/propidium iodide (PI) staining and FACS analysis (Figures 1a and b). Indeed, in contrast to its scrambled counterpart, TAT-Ctrl (Supplementary Figure 1), TAT-IDP^S^ triggered apoptotic cell death in four out of five DL-BCL cells (KARPAS422, TOLEDO, PFEIFFER, and SU-DHL-4), but not in OCI-LY-1. The latter was not due to a general defect in the apoptotic program in OCI-LY-1, because these cells were very sensitive toward more general apoptotic inducers, like staurosporine (Figure 1c).

### TAT-IDP^S^ effectively provokes cell death in SU-DHL-4 but not in OCI-LY-1

**Figure 2:**
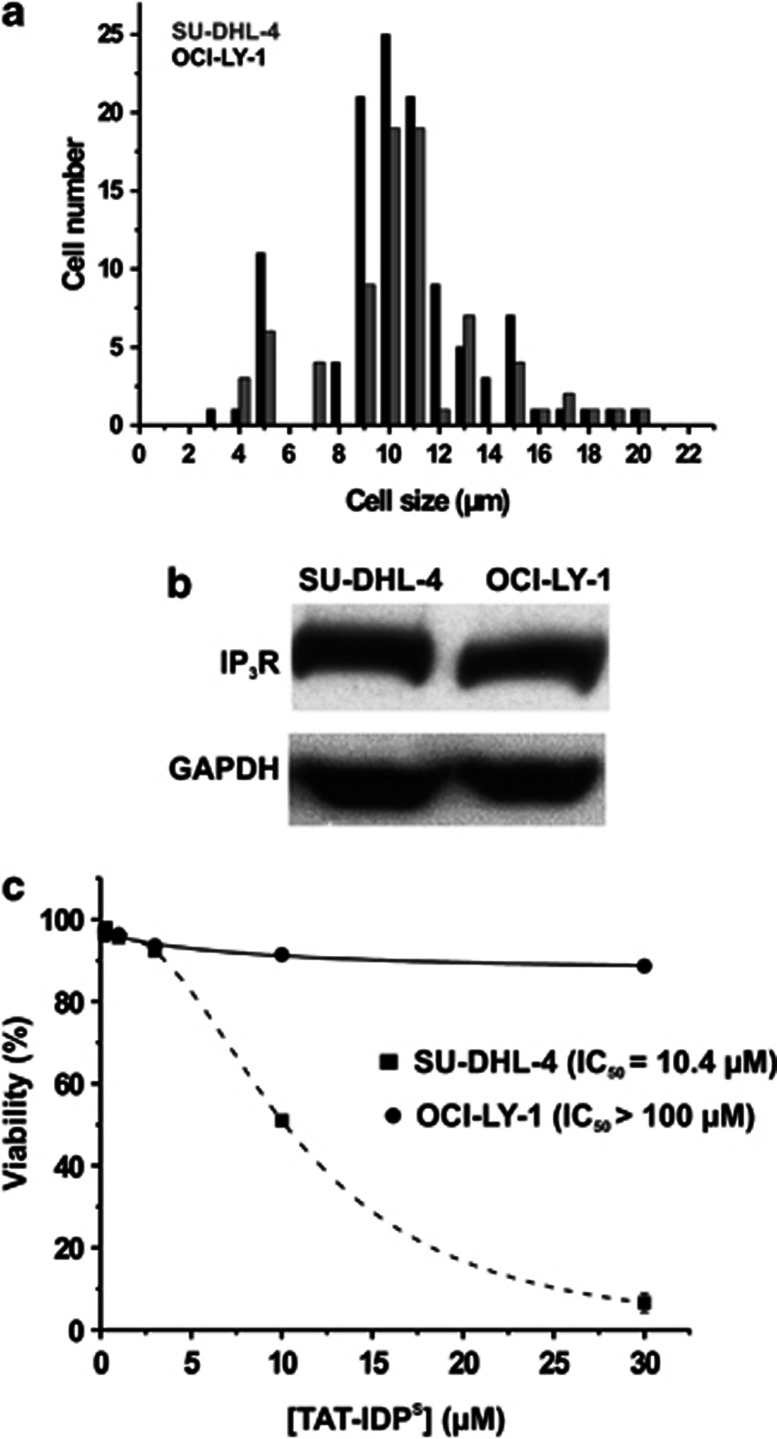
SU-DHL-4 and OCI-LY-1 cells have differential TAT-IDP^S^-induced cell death. (**a**) Average-size measurement of SU-DHL-4 and OCI-LY-1 viable cells with a Tali Image-Based Cytometer using trypan-blue staining. Average viable-cell size for OCI-LY-1 is 11.2 *μ*m and for SU-DHL-4 is 11.5 *μ*m. (**b**) A representative western blot showing the IP_3_R-expression level in SU-DHL-4 and OCI-LY-1. The expression level of GAPDH protein was used as control for equal loading. (**c**) Dose-response curves comparing viability of SU-DHL-4 *versus* OCI-LY-1 based on flow cytometric quantification of PI exclusion 24 h after adding TAT-IDP^S^. Data are expressed as the average ±S.D.; *n*=4

Next, we decided to elucidate the underlying mechanisms for the different responses toward TAT-IDP^S^ treatment. We focused on comparing SU-DHL-4 and OCI-LY-1, because these cells are both germinal-center DL-BCL cells and are very similar in cell size (Figure 2a). Furthermore, both cell lines expressed similar total amounts of IP_3_R proteins (Figure 2b), whereas displaying the most divergent response to TAT-IDP^S^. We first determined a concentration-response curve for both cells toward TAT-IDP^S^-induced cell death (Figure 2c). We found that TAT-IDP^S^ killed SU-DHL-4 cells with an IC_50_ of about 10 *μ*M, whereas OCI-LY-1 cells were resistant to TAT-IDP^S^-induced cell death, even at 30 *μ*M, which killed about 90% of the SU-DHL-4 cells. Using FITC-labeled TAT-IDP^S^, we also confirmed that both cell lines accumulated the peptide to similar extents (Supplementary Figure 2).

### TAT-IDP^S^ triggers IP_3_R-mediated cytosolic [Ca^2+^] rises in SU-DHL-4 but not in OCI-LY-1

**Figure 3:**
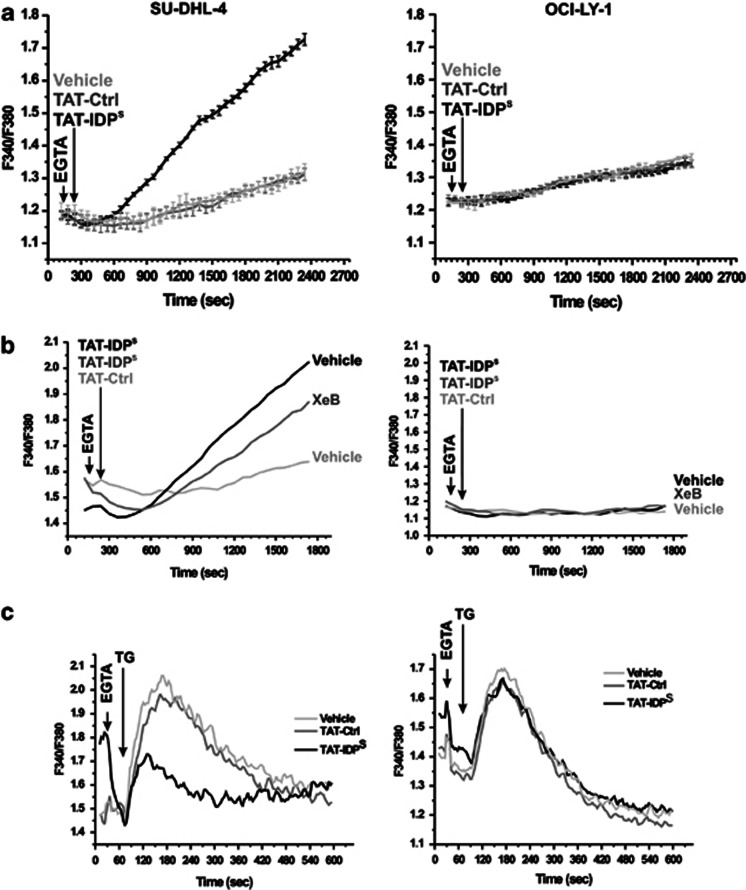
TAT-IDP^S^ triggers IP_3_R-mediated [Ca^2+^] rises in SU-DHL-4 but not in OCI-LY-1. (**a**) Representative traces from fluorimetric analysis of the TAT-IDP^S^-induced Ca^2+^ responses in SU-DHL-4 and OCI-LY-1 using the ratiometric Ca^2+^ indicator Fura2-AM. Vehicle solution and TAT-Ctrl were used as negative controls. The ratio of emitted fluorescence of Fura2 (F340/F380) was monitored and the three different treatments were added 60 s (second arrow) after the addition of 1 mM EGTA (first arrow). (**b**) Representative traces of Ca^2+^ responses induced by the treatment with 10 *μ*M TAT-IDP^S^ (second arrow) in SU-DHL-4 (left panel) and OCI-LY-1 (right panel) pretreated without (black traces) or with (gray traces) 10 *μ*M XeB for 30 min in the presence of 1 mM EGTA (first arrow). TAT-Ctrl was used as negative control (light gray traces). Recordings without XeB were exposed to the vehicle (dimethyl sulfoxide). (**c**) Analysis of the 10 *μ*M thapsigargin (TG)-induced Ca^2+^ responses in SU-DHL-4 and OCI-LY-1 pretreated without or with 10 *μ*M TAT-Ctrl or TAT-IDP^S^ for 30 min. The curves are representative of three independent experiments

**Figure 4:**
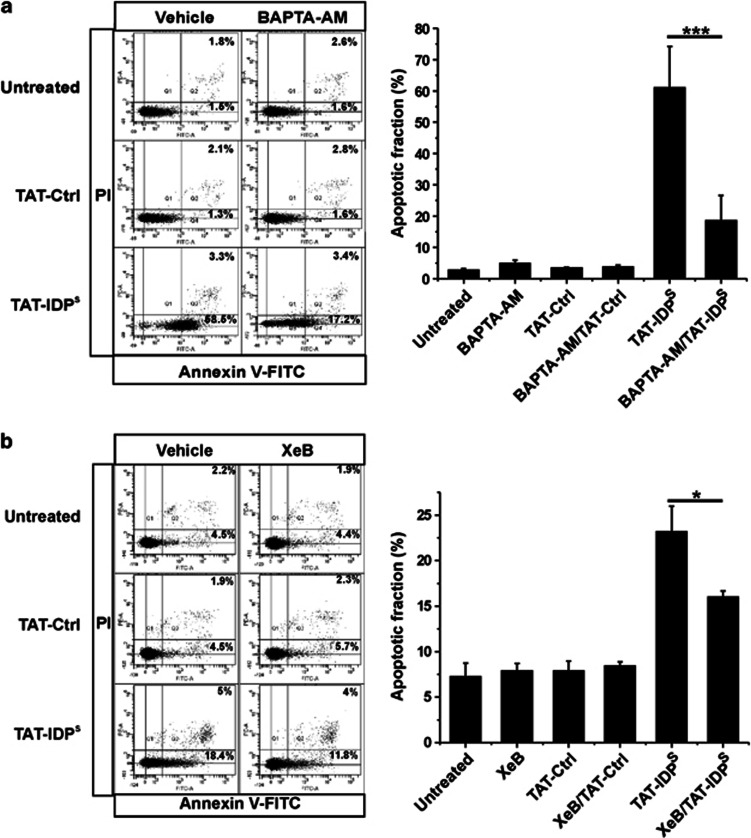
TAT-IDP^S^ triggers IP_3_R-mediated apoptosis in SU-DHL-4. (**a**) Dot plots from flow-cytometry analysis of apoptosis induced by the treatment without or with 10 *μ*M TAT-Ctrl or 10 *μ*M TAT-IDP^S^ in SU-DHL-4 in the presence or absence of 10 *μ*M BAPTA-AM for 2 h. (**b**) Dot plots from flow cytometry analysis of apoptosis induced by the treatment without or with 5 *μ*M TAT-Ctrl or 5 *μ*M TAT-IDP^S^ in SU-DHL-4 pretreated without or with 2.5 *μ*M XeB for 2 h. A quantitative analysis of four independent experiments with the apoptotic population identified as the annexin V-FITC-positive fraction (Q2+Q4) of each condition is shown in the right panel. Data were calculated and shown as average±S.D. Statistically significant differences are labeled with * (*P*<0.05) using a Student's *t*-test (paired two-tailed)

Next, we monitored cytosolic Ca^2+^ signals in response to acute TAT-IDP^S^ exposure in a Ca^2+^-free extracellular medium (Figure 3). After Fura2-AM loading of SU-DHL-4 and OCI-LY-1, cytosolic [Ca^2+^] measurements were performed in cell populations using an automated fluorescence plate reader. We found that TAT-IDP^S^ (10 *μ*M), but not TAT-Ctrl, caused an accelerated increase in the cytosolic [Ca^2+^] in SU-DHL-4, whereas this was not observed in OCI-LY-1 (Figure 3a). The increase in cytosolic [Ca^2+^] could be counteracted by using the selective IP_3_R antagonist xestospongin B (XeB),^27^ indicating a major role for IP_3_Rs in mediating the TAT-IDP-induced [Ca^2+^] rise in SU-DHL-4 (Figure 3b). Indeed, XeB reduced the slope of the TAT-IDP^S^-induced [Ca^2+^] rise by about 40%. These observations were underpinned by additional experiments in which the ER Ca^2+^ content was assessed using 10 *μ*M thapsigargin, a potent and selective inhibitor of the ER Ca^2+^ ATPases (SERCA)^28^ together with EGTA for chelating extracellular Ca^2+^. The magnitude of the thapsigargin-induced [Ca^2+^] rise (area under the peak) is a measure of the amount of Ca^2+^ stored in the ER. We found that pretreating the cells with TAT-IDP^S^, but not TAT-Ctrl, severely reduced the thapsigargin-induced [Ca^2+^] rise in SU-DHL-4. In contrast, TAT-IDP^S^ pretreatment had only a slight effect on the thapsigargin-induced [Ca^2+^] rise in OCI-LY-1 as compared with SU-DHL-4 (Figure 3c). Importantly, all these measurements were done well before apoptotic cell death was observed, indicating that the acute rise of [Ca^2+^] was not due to ongoing apoptotic processes, but was rather a very proximal event in the induction of apoptosis. This is supported by the fact that treating SU-DHL-4 with BAPTA-AM (10 *μ*M), a cell-permeable Ca^2+^ chelator, reduced TAT-IDP^S^-induced cell death by ∼75% (Figure 4a). Similar results were observed for XeB (2.5 *μ*M), which inhibits IP_3_R-mediated Ca^2+^ release. Indeed, a 2-h pre-treatment of SU-DHL-4 with XeB reduced the number of apoptotic cells (i.e., annexin V-FITC-positive cells) in response to TAT-IDP^S^ by about 40% in comparison to SU-DHL-4 treated with TAT-IDP^S^ alone (Figure 4b). This correlates with XeB's inhibitory effect on TAT-IDP^S^-induced [Ca^2+^] rise (Figure 3b). XeB at 2.5 *μ*M did not completely prevent apoptosis, probably because IP_3_R signaling was not completely blocked in these experimental conditions. Higher concentrations of XeB could, however, not be used because they seemed toxic to these cells (data not shown). This effect may be related to the important role of IP_3_Rs in autophagy, which is important for cancer cell survival.^29, 30^

### Both SU-DHL-4 and OCI-LY-1 display upregulated Bcl-2, but express different IP_3_R isoforms

**Figure 5:**
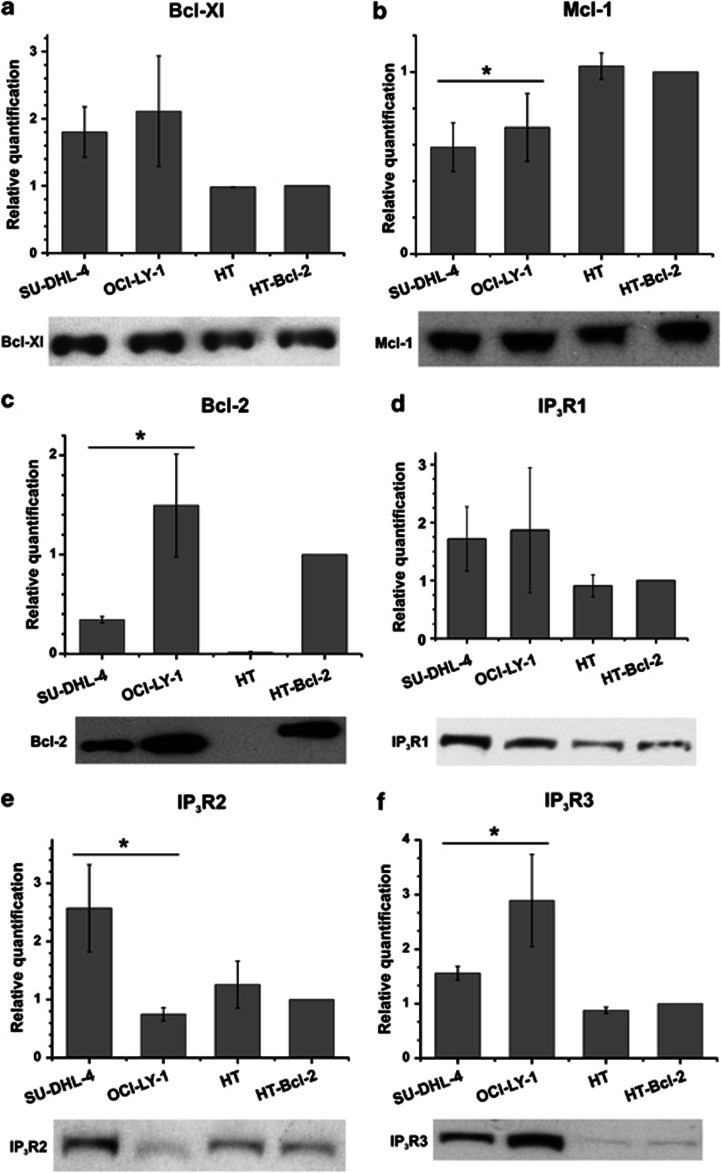
Analysis of anti-apoptotic Bcl-2-family members and IP_3_R isoform-expression levels in SU-DHL-4, OCI-LY-1, HT, and HT-Bcl-2. qPCR and western blot were used to analyze, respectively, the mRNA- and protein-expression levels of (**a**) Bcl-Xl, (**b**) Mcl-1, (**c**) Bcl-2, (**d**) IP_3_R1, (**e**) IP_3_R2, and (**f**) IP_3_R3 in SU-DHL-4, OCI-LY-1, HT, and HT-Bcl-2. A quantification relative to HT-Bcl-2 was done for qPCR data and shown as average±S.D. Statistically significant differences between expression in SU-DHL-4 and OCI-LY-1 are labeled with *(*P*<0.05, using a Student's paired two-tailed *t*-test)

We quantified the expression levels of a variety of anti-apoptotic Bcl-2-family members and IP_3_R isoforms in both SU-DHL-4 and OCI-LY-1 at the mRNA level using specific probes (Figure 5, bar graphs), and at the protein level using specific and validated antibodies (Figure 5, blots). As reference cell lines, we used HT cells, a DL-BCL cell line, which has very low endogenous levels of Bcl-2, and HT cells ectopically and stably overexpressing Bcl-2 (HT-Bcl-2). We found that both SU-DHL-4 and OCI-LY-1 displayed similar levels of Bcl-Xl (Figure 5a) and Mcl-1 (Figure 5b). These levels were also similar to the levels found in HT and HT-Bcl-2, although Bcl-Xl was slightly higher in both SU-DHL-4 and OCI-LY-1. For Bcl-2, we also found a high expression in both SU-DHL-4 and OCI-LY-1. Its level was in the range of the Bcl-2-overexpressing HT (Figure 5c). However, Bcl-2 levels were significantly higher in OCI-LY-1 than in SU-DHL-4.

We also probed IP_3_R isoform-expression levels by qRT-PCR and by western blot analysis using isoform-specific antibodies. We also found that both SU-DHL-4 and OCI-LY-1 displayed similar levels of IP_3_R1, which were slightly higher than the ones observed in HT and HT-Bcl-2 (Figure 5d). However, SU-DHL-4 and OCI-LY-1 displayed a very different profile for IP_3_R2 (Figure 5e) and IP_3_R3 (Figure 5f). Indeed, SU-DHL-4 displayed a strong upregulation of IP_3_R2-mRNA and -protein levels as compared with the other cell lines, whereas OCI-LY-1 displayed the highest IP_3_R3-mRNA and -protein levels.

### TAT-IDP^s^-induced apoptosis is suppressed in SU-DHL-4 by IP_3_R2 knockdown and boosted in OCI-LY-1 by IP_3_R2 overexpression

Bcl-2 levels were higher in OCI-LY-1 than in SU-DHL-4 (Figure 5c). Thus, we examined whether the resistance of OCI-LY-1 to TAT-IDP^S^-induced apoptosis was due to a higher anti-apoptotic Bcl-2 reserve of these cells. Using two independent siRNA probes (siBcl-2(1) and siBcl-2(2)), we successfully knocked down Bcl-2 in OCI-LY-1 by about 30% and 90%, respectively, in comparison to mock- or siCtrl-transfected OCI-LY-1, while not affecting Bcl-Xl levels (Supplementary Figure 3, blots). Strikingly, Bcl-2 knockdown did not significantly increase the apoptotic response toward TAT-IDP^S^ treatment (Supplementary Figure 3, bottom panel). This result showed that the higher expression of Bcl-2 in OCI-LY-1 in comparison to SU-DHL-4 was not responsible for the resistance of OCI-LY-1 to TAT-IDP^S^-induced apoptosis.

**Figure 6:**
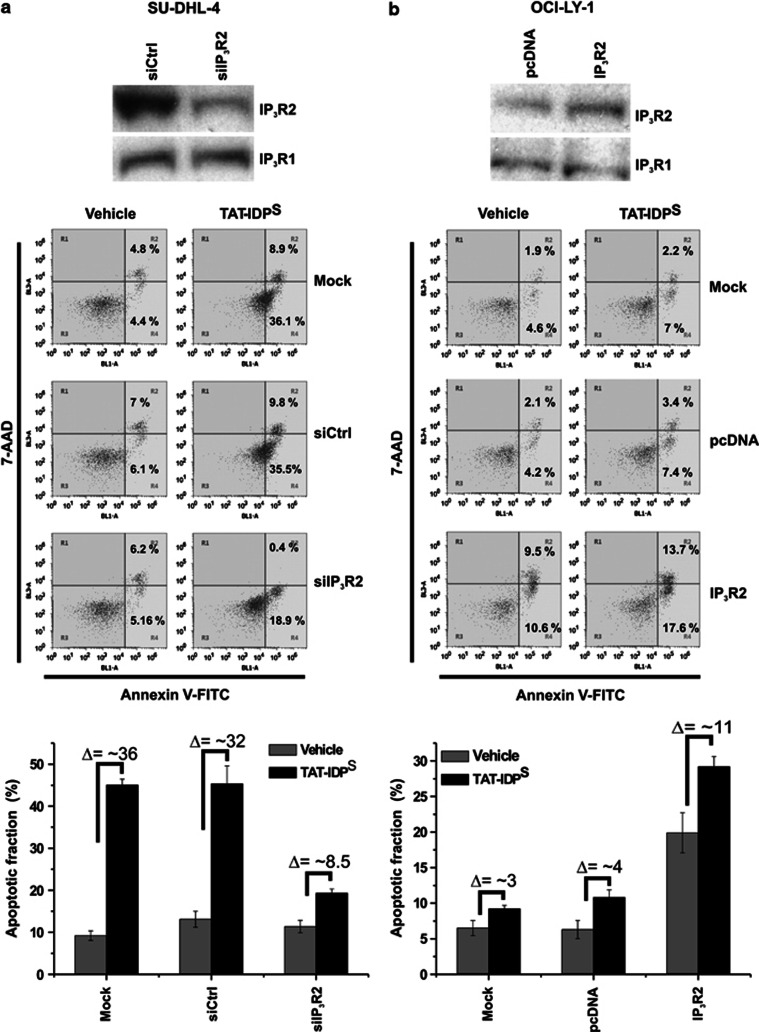
Manipulation of IP_3_R2-expression levels in OCI-LY-1 and SU-DHL-4. (**a**) Western-blot analysis of IP_3_R2 and IP_3_R1 proteins in siCtrl- and siIP_3_R2-transfected SU-DHL-4 is shown in the upper panel. Representative dot plots from flow cytometry analysis of apoptosis induced by 2 h treatment without or with 10 *μ*M TAT-IDP^S^ in mock-, siCtrl-, and siIP_3_R2-transfected SU-DHL-4 are shown in the middle panel. Quantitative analysis of three independent experiments of the TAT-IDP^S^-induced apoptosis is shown in the bottom panel. (**b**) Western-blot analysis of IP_3_R2 and IP_3_R1 proteins in empty vector- and IP_3_R2 expressing plasmid-transfected OCI-LY-1 is shown in the upper panel. Representative dot plots from flow cytometry analysis of apoptosis induced by 2 h treatment without or with 10 *μ*M TAT-IDP^S^ in mock-, empty vector-, and IP_3_R2-expressing plasmid-transfected OCI-LY-1 cells are shown in the middle panel. Quantitative analysis of three independent experiments of the TAT-IDP^S^-induced apoptosis is shown in the bottom panel. Results are shown as average±S.D.

Given the striking difference in IP_3_R-expression profile and the distinct sensitivity of these isoforms toward IP_3_, we wondered whether high IP_3_R2 expression was underlying the sensitivity of DL-BCL cells toward TAT-IDP^S^ exposure. Importantly, IP_3_R2 and IP_3_R3 have very distinct ligand sensitivity: IP_3_R2 is the IP_3_R isoform most sensitive toward IP_3_, whereas IP_3_R3 is the IP_3_R isoform least sensitive toward IP_3_.^31^ Therefore, we developed a siRNA probe selectively targeting IP_3_R2 and a non-targeting control siRNA probe (siCtrl). This siRNA probe (siIP_3_R2) effectively knocked down IP_3_R2-protein levels in transfected SU-DHL-4 cells by about 60% in comparison to mock-transfected or siCtrl-transfected SU-DHL-4 cells (Figure 6a, blots). IP_3_R2 knockdown correlated with an increased resistance toward TAT-IDP^S^ treatment (Figure 6a, bottom panels). Indeed, the siIP_3_R2 probe significantly reduced the number of apoptotic cells in TAT-IDP^S^-treated SU-DHL-4 in comparison to mock-transfected or siCtrl-transfected SU-DHL-4 (Figure 6a, bottom panel). Conversely, when we increased the expression of IP_3_R2 in OCI-LY-1 cells by the transfection of an IP_3_R2-expression plasmid, OCI-LY-1 became more sensitive toward apoptotic cell death. Transfection with the plasmid increased the IP_3_R2-protein level in OCI-LY-1 by more than 50% in comparison to empty vector-transfected OCI-LY-1 (Figure 6b, blots). The exogenous IP_3_R2 expression significantly increased the spontaneous apoptosis in the transfected cells from ∼5 to ∼20%. It also increased the number of apoptotic cells in TAT-IDP^S^-treated OCI-LY-1 cells in comparison to mock-transfected or empty vector-transfected OCI-LY-1 from ∼10 to 30% (Figure 6b, bottom panels).

### IP_3_R2-protein levels correlate with the sensitivity toward the TAT-IDP^S^-induced apoptotic [Ca^2+^] rise in DL-BCL cells

**Figure 7:**
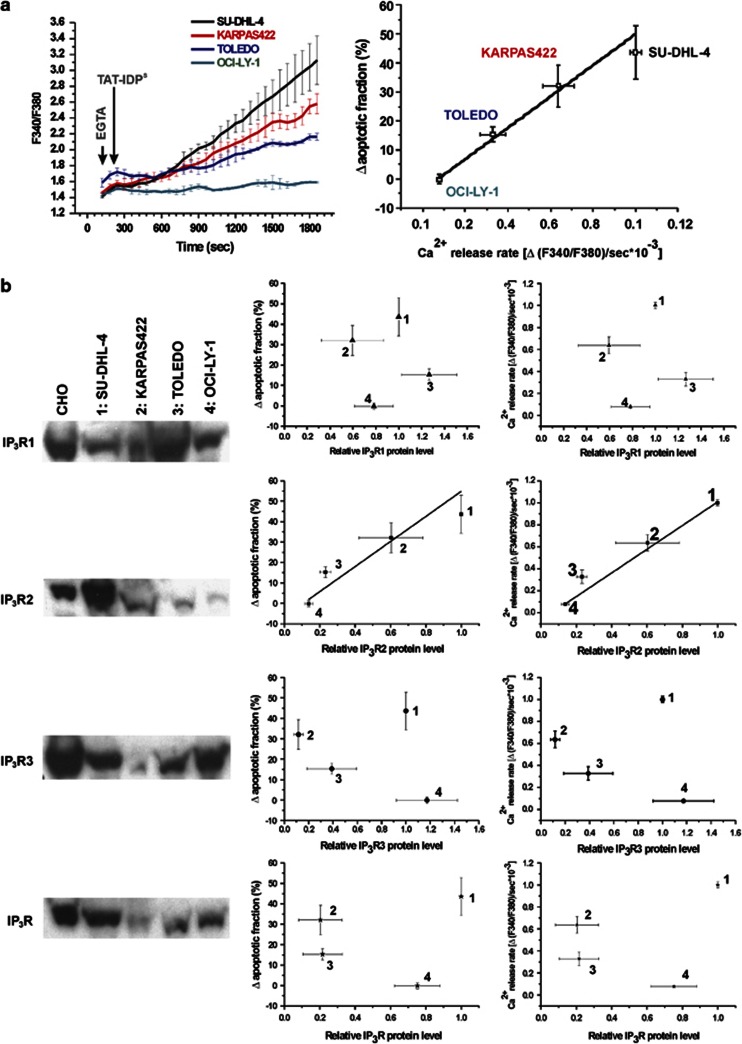
TAT-IDP^S^-induced apoptosis depends on the IP_3_R2-expression level. (**a**) Left panel: representative traces from fluorimetric analysis of the TAT-IDP^S^-induced Ca^2+^ responses in SU-DHL-4, KARPAS422, TOLEDO, and OCI-LY-1 using the ratiometric Ca^2+^ indicator Fura2-AM in the presence of 1 mM EGTA. Right panel: linear fitting of the TAT-IDP^S^-induced apoptosis identified as the annexin V-FITC-positive fraction as a function of the slope of the [Ca^2+^] rises induced by TAT-IDP^S^ for SU-DHL-4, KARPAS422, TOLEDO, and OCI-LY-1. (**b**) Left panel: Western blots analyzing the protein expression levels of IP_3_R1, IP_3_R2, IP_3_R3, and total IP_3_R in SU-DHL-4, KARPAS422, TOLEDO, and OCI-LY-1. Microsomes from CHO cells were used as a standard positive control for protein quantification. The blots are representative of more than four independent experiments. Central panel: linear fitting of the TAT-IDP^S^-induced apoptosis as a function of the IP_3_R1, IP_3_R2, IP_3_R3, and total IP_3_R relative protein levels in SU-DHL-4 (1), KARPAS422 (2), TOLEDO (3), and OCI-LY-1 (4). The levels are expressed relative to the level in SU-DHL-4. Right panel: linear fitting of the TAT-IDP^S^-induced Ca^2+^ response as a function of the IP_3_R1, IP_3_R2, IP_3_R3, and total IP_3_R relative protein levels in SU-DHL-4 (1), KARPAS422 (2), TOLEDO (3), and OCI-LY-1 (4)

**Figure 8:**
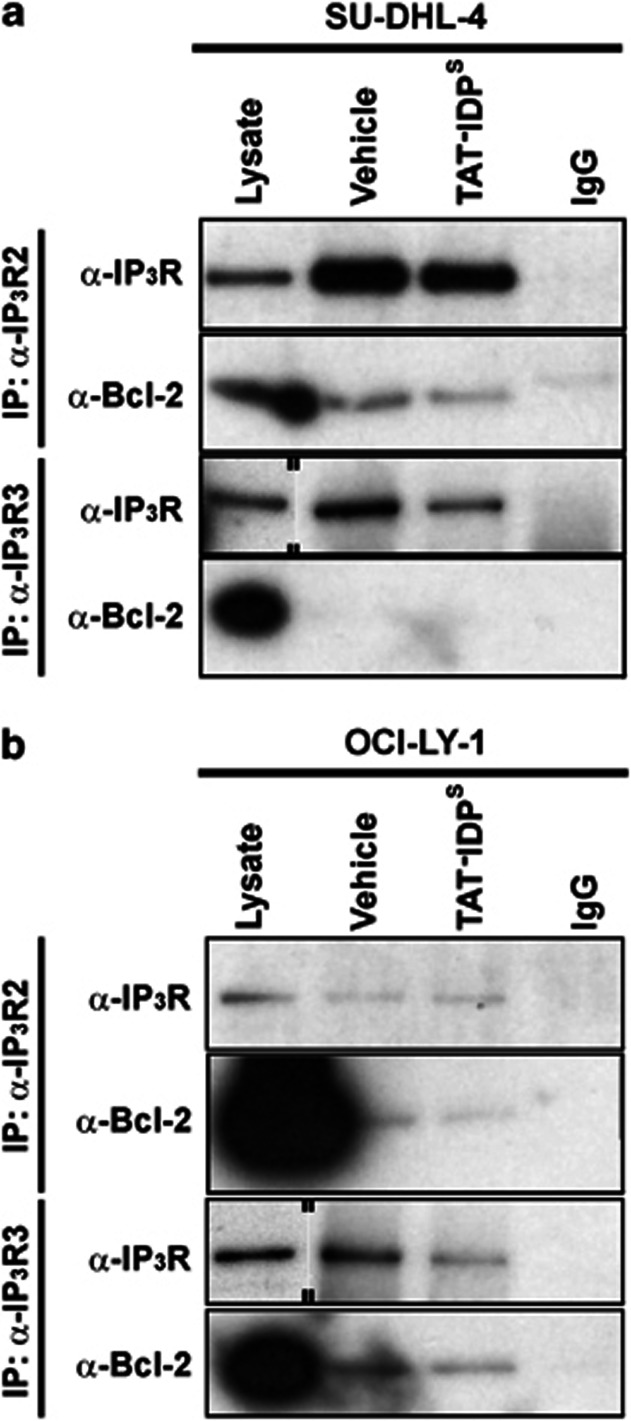
TAT-IDP^S^ disturbs Bcl-2/IP_3_R complexes. Representative immunoprecipitation (IP_3_R2 and IP_3_R3) and co-immunoprecipitation experiment of Bcl-2 with IP_3_R2 and IP_3_R3 from lysates of (**a**) SU-DHL-4 and (**b**) OCI-LY-1 pretreated for 2 h without or with 10 *μ*M TAT-IDP^S^. IgG=negative control and lysate=positive control. The blots are representative of three independent experiments. The double lines on the blots indicate that lanes from another part of the same gel and exposure time were merged

Next, we monitored cytosolic Ca^2+^ signals in response to acute TAT-IDP^S^ exposure in the Bcl-2-dependent cell lines (Figure 7). After Fura2-AM loading of SU-DHL-4, KARPAS422, TOLEDO, and OCI-LY-1, cytosolic [Ca^2+^] measurements were performed in cell populations. We found that TAT-IDP^S^ (10 *μ*M) caused a differential increase in cytosolic [Ca^2+^] in the different DL-BCL cell lines (Figure 7a, left panel). We plotted the slope of the cytosolic [Ca^2+^] increase as a function of the TAT-IDP^S^-induced apoptosis in the different DL-BCL cell lines. A positive linear correlation (*r*^2^=0.97) existed between the TAT-IDP^S^-induced [Ca^2+^] rise and its apoptotic effect (Figure 7a, right panel). We also performed an IP_3_R-profile analysis for the four DL-BCL cell lines using a pan-IP_3_R antibody recognizing all three IP_3_R isoforms and using isoform-specific antibodies (Figure 7b, blots, left panels). Plotting the apoptotic responses (Figure 7b, central panels) and [Ca^2+^] responses (Figure 7b, right panels) to TAT-IDP^S^ as a function of the different IP_3_R isoforms and total IP_3_R-protein levels for the different DL-BCL cell lines revealed that only IP_3_R2-protein levels, but not IP_3_R1, IP_3_R3, nor total IP_3_R, correlated with TAT-IDP^S^-induced apoptosis (*r*^2^=0.7) or with TAT-IDP^S^-induced [Ca^2+^] rises (*r*^2^=0.99). TOLEDO and KARPAS422 displayed intermediate IP_3_R2-protein levels, correlating with intermediate apoptotic and Ca^2+^ responses to TAT-IDP^S^ exposure. In a next series of experiments, we examined whether TAT-IDP^S^ differently affected IP_3_R/Bcl-2 complexes in SU-DHL-4 and OCI-LY-1 (Figures 8a and b). We have previously shown that the Bcl-2-binding site is conserved among all three IP_3_R isoforms.^22^ At least in *in vitro* surface–plasmon–resonance experiments, recombinantly expressed and purified fragments covering the proposed Bcl-2-binding site of IP_3_R1, IP_3_R2, and IP_3_R3 were able to interact with the synthetic BH4 domain of Bcl-2.^22^ Thus, we examined whether this was also valid in a cellular context, and whether Bcl-2 co-immunoprecipitated with IP_3_Rs from SU-DHL-4 and OCI-LY-1 cell lysates. Immunoprecipitation of IP_3_R2 indeed caused the co-immunoprecipitation of Bcl-2 in both SU-DHL-4 and OCI-LY-1 lysates. However, despite the fact that OCI-LY-1 displayed higher levels of Bcl-2 than SU-DHL-4, the amount of Bcl-2 that was specifically co-immunoprecipitated with IP_3_R2 in OCI-LY-1 was extremely low. Importantly, we found that pretreatment of SU-DHL-4 with TAT-IDP^S^ reduced the amount of Bcl-2 co-immunoprecipitating with IP_3_R2 (Figure 8a). A similar band was observed in OCI-LY-1, but due to the much lower levels of Bcl-2 binding to IP_3_R2 it was just above the detection level and this was despite the very high Bcl-2 levels in these cells. For IP_3_R3, we found that only in OCI-LY-1, but not in SU-DHL-4, Bcl-2 co-immunoprecipitated with IP_3_R3. Pretreatment with TAT-IDP^S^ only slightly reduced Bcl-2 levels in the IP_3_R3 co-immunoprecipitated samples (Figure 8b). Hence, these experiments indicate that in SU-DHL-4 Bcl-2 was recruited to a large extent by IP_3_R2, and Bcl-2 could be displaced at least partially from this isoform using TAT-IDP^S^. This was not observed in OCI-LY-1 with respect to the predominant IP_3_R3 isoform in these cells. This could mean that the Bcl-2/IP_3_R3 interaction is less pronounced in a cellular context or alternatively that Bcl-2 in these cells is mainly bound to other proteins such as Bim and Bax.^12^ Thus, these observations suggest that the TAT-IDP^S^-induced [Ca^2+^] rise and cell death are linked to the disruption of the IP_3_R/Bcl-2 interaction, particularly in cells expressing relatively high levels of IP_3_R2.

## Discussion

The major findings of this study are that (i) IP_3_R2 is a determinant of the sensitivity of Bcl-2-dependent ‘primed to death' DL-BCL cells toward the apoptotic effect of TAT-IDP^S^, and (ii) Bcl-2-dependent cancer cells may be addicted to high levels of Bcl-2 to suppress aberrant pro-apoptotic Ca^2+^ signals. In particular, cancer cells expressing the most sensitive IP_3_R isoform (IP_3_R2) likely are very vulnerable toward tonic IP_3_ signaling.

### Peptide tools selectively targeting BH4-Bcl-2 are effective in DL-BCL cancer cells expressing high levels of IP_3_R2

Our study is the first to provide a prominent role for distinct IP_3_R isoforms in cell death and survival processes in malignant cells. The higher IP_3_ sensitivity of IP_3_R2 could render cells sensitive to very low levels of IP_3_. In that respect, TAT-IDP^S^ may trigger Ca^2+^-release events by disrupting Bcl-2/IP_3_R2 interactions, in conditions of low-level stimulation and close to basal cellular IP_3_ concentrations. These events may not be sufficient to trigger activation of the least sensitive IP_3_R isoform, the IP_3_R3. This would render cancer cells expressing mainly IP_3_R3 resistant to TAT-IDP^S^. At the molecular level, the sensitivity toward TAT-IDP^S^ is reflected in the presence of different Bcl-2/protein complexes. Indeed, the very sensitive SU-DHL-4 displayed high levels of IP_3_R/Bcl-2-complex formation, whereas this was not the case for the resistant OCI-LY-1, although this cell line expressed even higher levels of Bcl-2 than SU-DHL-4. Our observation is fully in line with a previous report showing that OCI-LY-1 displayed high levels of Bcl-2/Bax complex formation, which was not the case for SU-DHL-4.^12^ Hence, it seems that dependent on the apoptotic escape route cells may be addicted to high levels of Bcl-2 either to suppress aberrant IP_3_R activity (like in the case of IP_3_R2-expressing cancer cells, e.g., SU-DHL-4) or to suppress aberrant Bax activity (like in the case of IP_3_R3-expressing cancer cells, e.g., OCI-LY-1). Indeed, although both IP_3_R isoforms may interact with Bcl-2 *in vitro*, the occurrence and significance of these interactions may be very different in a cellular context. Therefore, it may be less critical for cancer cells to use Bcl-2 for suppressing the activity of IP_3_R3, because this isoform is the least sensitive to IP_3_ and thus to ongoing B-cell receptor (BCR) signaling. In contrast, cancer cells expressing high levels of IP_3_R2 will be addicted to high levels of Bcl-2 to suppress the pro-apoptotic activity of the hypersensitive IP_3_R2 in response to ongoing IP_3_ signaling. Interestingly, it has been shown that DL-BCL cells have a chronically active BCR.^32^ Moreover, SU-DHL-4 and OCI-LY-1 are reported to have a similar moderate activation of PLC*γ*2.^33^ This may indicate that cancer cells may suppress the downstream effects of chronic BCR signaling by either Bcl-2/IP_3_R interactions to inhibit IP_3_R signaling or alternatively by switching to the less sensitive IP_3_R3 isoform. From our immunoprecipitation experiments, it was evident that TAT-IDP^S^ did not completely disrupt the binding of Bcl-2 to IP_3_Rs. This may be due to the fact that other Bcl-2 domains may contribute to IP_3_R binding.^34^ Yet, alternative mechanisms could be involved in the differential role of different IP_3_R isoforms in cell death. It has recently been shown that the phosphorylation of IP_3_R3 by Akt leads to diminished Ca^2+^ transfer to mitochondria and protection from apoptosis, suggesting an additional level of cell death regulation mediated by Akt.^35^ Therefore, we cannot exclude an implication of Akt-induced phosphorylation of IP_3_R3 in the resistance of cells that highly express IP_3_R3 (OCI-LY-1) toward TAT-IDP^S^ induction of apoptotic Ca^2+^ signals, rendering Bcl-2 proteins redundant for recruitment to the IP_3_R3 channels.

### Novel functions for IP_3_R2 in cancer cells beyond its canonical function in exocrine glands

This study also reveals a novel isoform-specific function for IP_3_R2. Although IP_3_R2 is expressed at very low levels in most tissues, it is highly expressed in organs with exocrine functions, correlating with its importance for the physiological exocrine function of these organs.^36^ IP_3_R2 cooperates with IP_3_R3 in nutrient digestion and enzymatic secretion, correlating with severely impaired Ca^2+^ signaling in double knock outs in acinar cells of the salivary glands and of the pancreas^37^ and in olfactory mucus secretion and function.^38^ In acinar cells, IP_3_R2 expression levels have been linked to the sensitivity toward metabolic stress, as IP_3_R2 is the most sensitive toward ATP regulation and determines the influence of ATP depletion on intracellular Ca^2+^ signaling.^39^ Here, we describe for the first time a prominent role for IP_3_R2 for the pathophysiology of B-cell lymphoma malignant cells. The aberrant IP_3_R2 upregulation in some B-cell cancer cells may be an additional component in their addiction to high levels of Bcl-2 to suppress toxic Ca^2+^ signals in response to chronic BCR signaling, adding another level of heterogeneity of these cancer cells toward dysregulation of apoptosis-signaling cascades. The mechanism underlying IP_3_R2 upregulation is not clear, but clearly is a transcriptionally regulated event. Also, the benefit for cancer cells to upregulate IP_3_R2 is not clear. Nevertheless, given the central role of constitutive IP_3_/Ca^2+^ signaling in regulating mitochondrial bio-energetics,^40^ IP_3_R2 upregulation may enhance mitochondrial function and energy production to accommodate for the higher metabolic activity and the induced proliferation of cancer cells.

## Conclusion

Our findings highlight the importance of targeting Bcl-2's BH4 domain in Bcl-2-dependent cancers. Although we previously showed that CLL may be targeted using IP_3_R-derived peptides, we now provide (i) evidence that this strategy is applicable in other cancer cells like DL-BCL, and (ii) mechanistic insights in the underlying signaling pathways revealing a prominent role for IP_3_R2. This strategy may be helpful to sensitize cancer cells to BH3-mimetic drugs, including cancer cells that are resistant to TAT-IDP^S^ itself. It also seems that exploiting the adaptive response of cancer cells toward higher metabolic needs putatively underlying IP_3_R2 upregulation may provide a novel way to target these cells through Ca^2+^-signaling dysregulation.

## Materials and Methods

### Cells

SU-DHL-4, KARPAS422, PFEIFFER, TOLEDO, HT, and HT-Bcl-2 (HT ectopically overexpressing Bcl-2) DL-BCL cell lines were cultured in suspension in RPMI-1640 media. The OCI-LY-1 DL-BCL cell line was cultured in suspension in Iscove modified Dulbecco medium (Invitrogen, Merelbeke, Belgium). All media were supplemented with 10% heat-inactivated fetal bovine serum, ℒ-glutamine (100 × GlutaMAX, Gibco/Invitrogen, 35050) and penicillin and streptomycin (100 × Pen/strep, Gibco/Invitrogen, 15070-063) at 37 °C and 5% CO_2_.

### Reagents

For immunoblot, antibodies were: anti-GAPDH (Sigma-Aldrich, Munich, Germany, G8795), anti-Bcl-Xl/S (Santa Cruz Biotechnologies, Heidelberg, Germany, l–19, sc-1041), anti-Mcl-1 (Santa Cruz Biotechnologies, s-19, sc819), and anti-Bcl-2 (Santa Cruz Biotechnologies, Franklin Lakes, NJ, USA, c-2, sc7382). Anti-IP_3_R1, anti-IP_3_R2, and anti-pan-IP_3_R were Rbt03,^41^ Rbt02^41^ and Rbt475^42^, respectively. Anti-IP_3_R3 was purchased from BD Biosciences (610312, Franklin Lakes, NJ, USA). For immunoprecipitations, antibodies against IP_3_R2 (Santa Cruz Biotechnologies, sc-7278) or IP_3_R3 (Santa Cruz Biotechnologies, sc-7277) were used. Other reagents include: Ca^2+^ ionophore A23187 (Sigma-Aldrich, C7522), EGTA (Acros Organics, Geel, Belgium, 409910250), thapsigargin (Enzo Life Sciences, Farmingdale, NY, USA, ALX-350-004-M010), ionomycin (LC Laboratories, Boston, MA, USA, I-6800), staurosporine (LC Laboratories, Kampenhout, Belgium, S-9300), and Fura2-AM (Biotium, Kampenhout, Belgium, 50033). XeB was purified from *Xestospongia exigua* as previously described.^27^ TAT-IDP^S^: RKKRRQRRRGGNVYTEIKCNSLLPLAAIVRV and TAT-Ctrl: RKKRRQRRRGGSIELDDPRPR were purchased from LifeTein (South Plainfield, New Jersey, USA) (purity>85%). The plasmid for IP_3_R2 expression was provided by Dr. Mikoshiba.^43, 44^

### Real-time qPCR

Total cellular RNA was isolated using the High Pure RNA Isolation Kit (Roche, Basel, Switzerland, 11 828 665 001). For cDNA synthesis, 1 *μ*g of RNA was reverse transcribed with High Capacity cDNA Reverse Transcription kit (Applied Biosystems, Foster City, CA, USA). The cDNA was diluted 1 : 10 and the Taqman real-time qPCR analysis was performed with an Applied Biosystems 7900HT instrument using specific primers and fluorescent Taqman probes for *Bcl-Xl, Mcl-1, Bcl-2, IP*_*3*_*R1, IP*_*3*_*R2,* and *IP*_*3*_*R3* obtained from Integrated DNA Technology. The results were analyzed with SDS 2.3 and RQ manager software (Applied Biosystems), and expression of *Bcl-Xl*, *Mcl-1*, *Bcl-2*, *IP*_*3*_*R1*, *IP*_*3*_*R2* and *IP*_*3*_*R3* mRNA was determined relative to *GAPDH*. The data were collected from three separate experiments using quadruplicates. Sequences for qPCR primers and probes are given as Supplementary Table 1.

### siRNA transfection

Sequences for siRNAs were: siIP_3_R2: 5′-GAAUGCCUAUAACCAAGGAdTdT-3′, siCtrl: 5′-GCGACCAACCGCATCTTAAdTdT-3′, siBcl-2(1): 5′-GUACAUCCAUUAUAAGCUGdTdT-3′, siBcl-2(2): 5′-GGAGGAUUGUGGCCUUCUUUGAGdTdT-3′. DL-BCL cells were transfected by electroporation using AMAXA, Kit V (Lonza, Basel, Switzerland) Program P-005. Briefly, 5 × 10^6^ cells were transfected with 50 ng siIP_3_R2, 200 ng siBcl-2, or 2 *μ*g of the IP_3_R2 expressing plasmid. After transfection, cells were seeded at 1 × 10^6^ cells/ml and the change of gene expression was analyzed by western blot.

### Immunoprecipitation and western blotting

DL-BCL cells were washed with phosphate-buffered saline and incubated at 4 °C with 1 ml lysis buffer (25 mM HEPES, pH 7.5, 1% Triton X-100, 300 mM NaCl, 1.5 mM MgCl_2_, 10% glycerol, 20 mM *β*-glycerophosphate, 2 mM EDTA, 2 mM EGTA, 1 mM dithiothreitol, and protease inhibitors tablet (Roche, Basel, Switzerland)) for 30 min on an head-over-head rotor. Control microsomes were prepared from CHO cells as previously described.^45^ Cell lysates were centrifuged at 20 000 × *g* for 10 min at 4 °C. In the meantime, a nonspecific control antibody and the antibody against the appropriate IP_3_R isoform were purified by using the Antibody Clean-up Kit (Pierce, Rockford, IL, USA). Successively, 40 *μ*l of Protein-A/G Magnetic Beads (Biovision, Rockford, IL, USA) were rotated with 5 *μ*g of the antibodies for 15 min at room temperature and washed to remove the unbound antibodies. Subsequently, cell lysate (300 *μ*g protein) was added to the antibody-bound beads bringing to volume (500 *μ*l) with lysis buffer. After incubating overnight, the beads were washed five times with lysis buffer and boiled for 5 min in 30 *μ*l of lithium dodecyl sulfate sample buffer. The eluted proteins were resolved by SDS-PAGE and analyzed by western blotting as previously described.^22^

### Apoptosis assay

DL-BCL cells were treated at 5 × 10^5^ cells/ml, pelleted by centrifugation, and incubated with annexin V-FITC (Becton Dickinson, Franklin Lakes, NJ, USA, 556419) and PI (Sigma, P4864) or 7-AAD (Becton Dickinson, 555815). Cell suspensions were analyzed by FACSCanto (Becton Dickinson) or Attune (Applied Biosystems). Cell death by apoptosis was scored by quantifying the population of annexin V-FITC-positive cells. Flow cytometric data were plotted and analyzed using BD FACSDiva Software (Becton Dickinson) or Attune version 2.1.0 (Applied Biosystems).

### Fluorescence Ca^2+^ measurements in intact cells

For the Ca^2+^ measurements in intact cells, DL-BCL cells were seeded in poly-ℒ-lysine-coated 96-well plates (Greiner) at a density of approximately 5 × 10^5^ cells/ml. The cells were loaded for 30 min with 5 *μ*M Fura2-AM at 25 °C in modified Krebs solution. They were then further incubated for at least 30 min without Fura2-AM. Fluorescence was monitored on a FlexStation 3 microplate reader (Molecular Devices, Sunnyvale, CA, USA) by alternately exciting the Ca^2+^ indicator at 340 and 380 nm and collecting emission fluorescence at 510 nm.

### Statistical analysis

Results are expressed as average±S.D., and *n* refers to the number of independent experiments. Significance was determined using a two-tailed paired Student's *t*-test. Differences were considered significant at *P*<0.05.

## Abbreviations

Bcl-2: B-cell lymphoma 2
Bcl-Xl: B-cell lymphoma-extra large
BCR: B-cell receptor
CLL: chronic lymphocytic leukemia
DL-BCL: diffuse large B-cell lymphoma
ER: endoplasmic reticulum
IP_3_: inositol 1,4,5-trisphosphate
IP_3_R: IP_3_ receptor
Mcl-1: myeloid-cell leukemia 1
TAT-IDP^S^: stabilized TAT-fused IP_3_R-derived peptide
XeB: xestospongin B
