# Supplementary material for: IP3R2 levels dictate the apoptotic sensitivity of diffuse large B-cell lymphoma cells to an IP3R-derived peptide targeting the BH4 domain of Bcl-2
Source: Cell Death Dis. 2013 May 16;4(5):e632–. doi: 10.1038/cddis.2013.140 (PMC3674349; doi:10.1038/cddis.2013.140)
Supplement: Supplementary data [file cddis2013140x1.doc]

**Supplemental data**

**Methods**

|  | 5’- primer 1 -3’ | 5’- primer 2 -3’ | Probe  5’- /56-FAM/Sequence/3IABkFQ/ -3’ |
| --- | --- | --- | --- |
| Bcl-Xl | CTCGTCTCTGGTTAGTGATTCTC | CCTCTCCCGACCTGTGATAC | CGGATTTGA/ZEN/ATCTCTTTCTCTCCCTTCAGA |
| Mcl-1 | ATATGCCAAACCAGCTCCTAC | AAGGACAAAACGGGACTGG | AGAACTCCA/ZEN/CAA |
| Bcl-2 | GCCAGGAGAGAAATCAAACAGAGG | GTGGATGACTGAGTACCTGAAC | CAGGATAAC/ZEN/GGAGGCTGGGATGC |
| IP_3_R1 | CTCCATCCTCAAATTCCACTTCA | CGACAGTGAAAACGCAGAGA | ACCAGTTCC/ZEN/TTGGGCCTCATGT |
| IP_3_R2 | ACCATCATCTCCACCCTCAT | AAGCAGACATGACAGTGAGAA | AGGCATTCT/ZEN/TCATCACATCCACCAGT |
| IP_3_R3 | AGCGATGCCTTATCATTGTAGA | CAAGTACGTCAAGAAGTGCCA | ACCTGCATT/ZEN/GGTCAGCTCAGTCAT |

**Table S1.** Sequences for qPCR primers and probes.

**Figure legends**

**Figure S1.** Flow-cytometry analysis of apoptosis using annexin V-FITC/PI-stained KARPAS422, OCI-LY-1, SU-DHL-4, TOLEDO and PFEIFFER untreated or treated with 10 µM TAT-Ctrl for 24 hrs (10,000 cells per analysis, representative experiment of three independent ones). The ∆ apoptotic fraction is a quantitative analysis of the TAT-IDP^S^-induced apoptosis (apoptotic population in treated cells (Q2 + Q4) – apoptotic population (Q2 + Q4) in untreated cells).

**Figure S2.** Histogram presentations of flow-cytometry analysis of FITC-TAT-IDP^S^ incorporation in SU-DHL-4 and OCI-LY-1. The full line represents the negative fluorescence limit corresponding to the untreated control.

**Figure S3.** siRNA-mediated knockdown of Bcl-2 has no effect on the sensitivity of OCI-LY-1 towards TAT-IDP^S^-induced apoptosis. A western-blot analysis of Bcl-2 and Bcl-Xl proteins in mock-, siCtrl-, siBcl-2(1)- and siBcl-2(2)-transfected OCI-LY-1 is shown in the upper panel. Representative dot plots from flow-cytometry analysis of apoptosis induced by 24 hrs treatment without or with 5 µM TAT-IDP^S^ in mock-, siCtrl- and two independent siBcl-2-transfected OCI-LY-1 cells are shown in the lower panel.
