# Supplementary figures and images for: IP3R2 levels dictate the apoptotic sensitivity of diffuse large B-cell lymphoma cells to an IP3R-derived peptide targeting the BH4 domain of Bcl-2
Source: Cell Death Dis. 2013 May 16;4(5):e632–. doi: 10.1038/cddis.2013.140 (PMC3674349; doi:10.1038/cddis.2013.140)

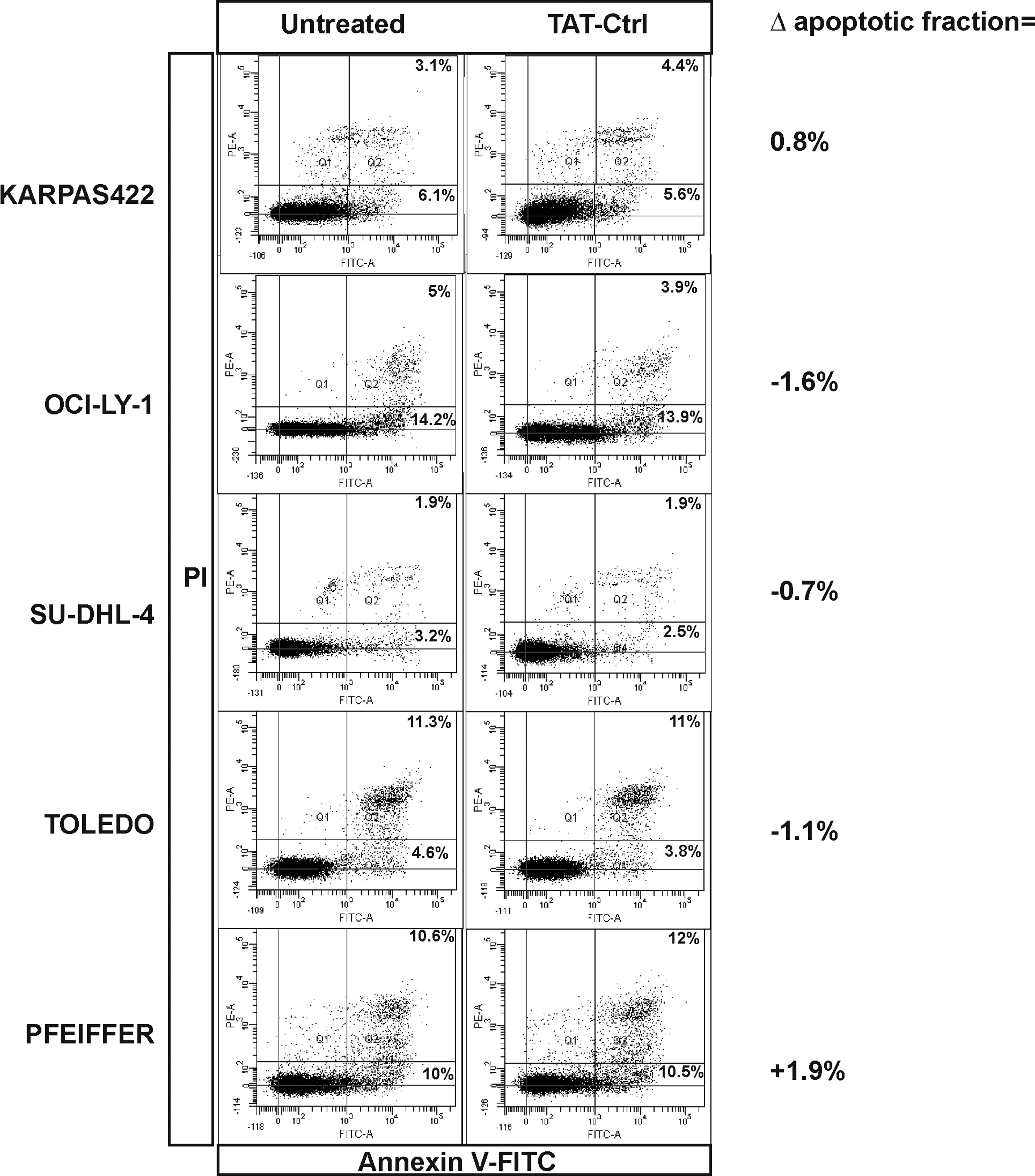

Supplement: Supplementary Figure S1 [file cddis2013140x2.tif]

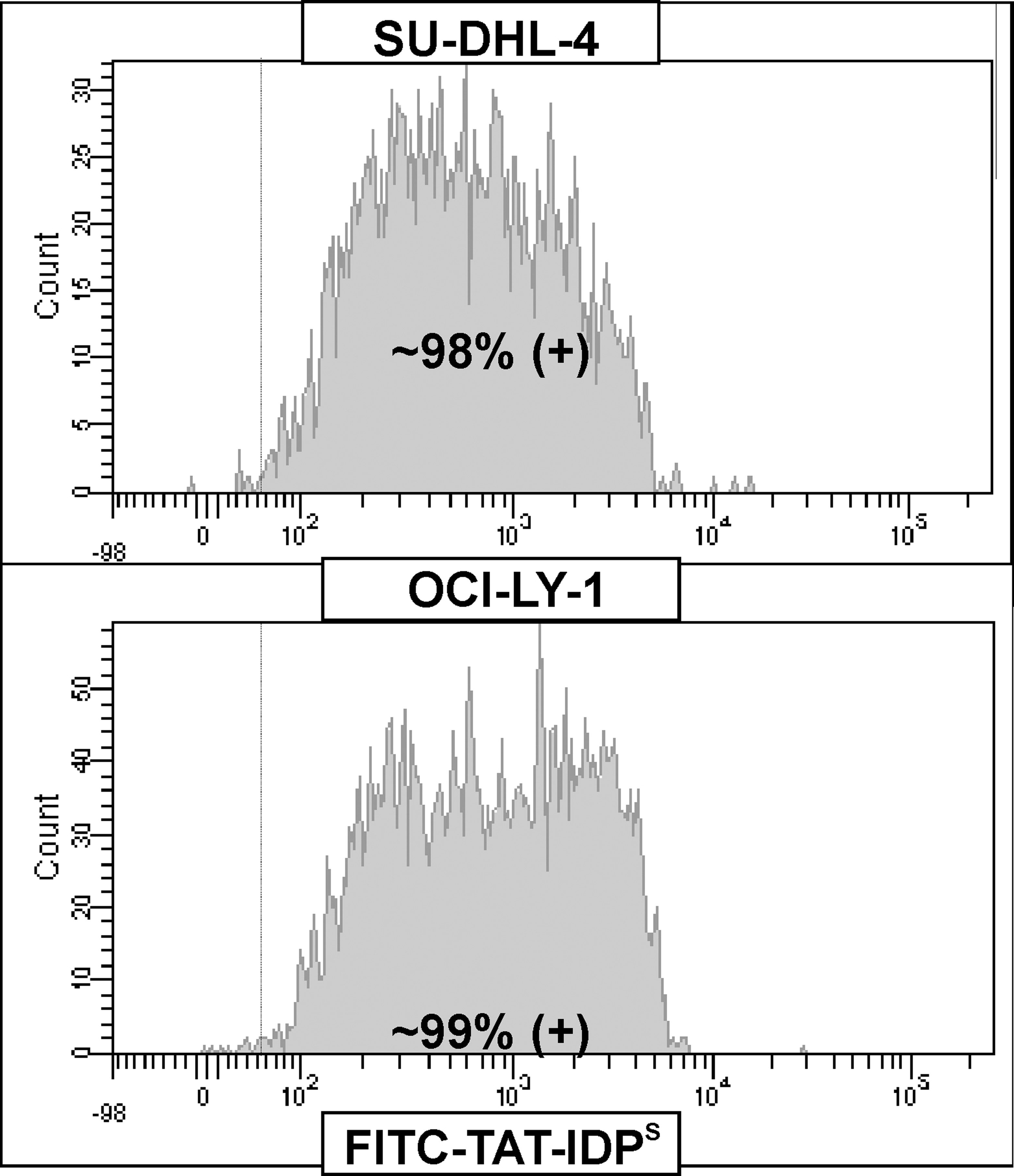

Supplement: Supplementary Figure S2 [file cddis2013140x3.tif]

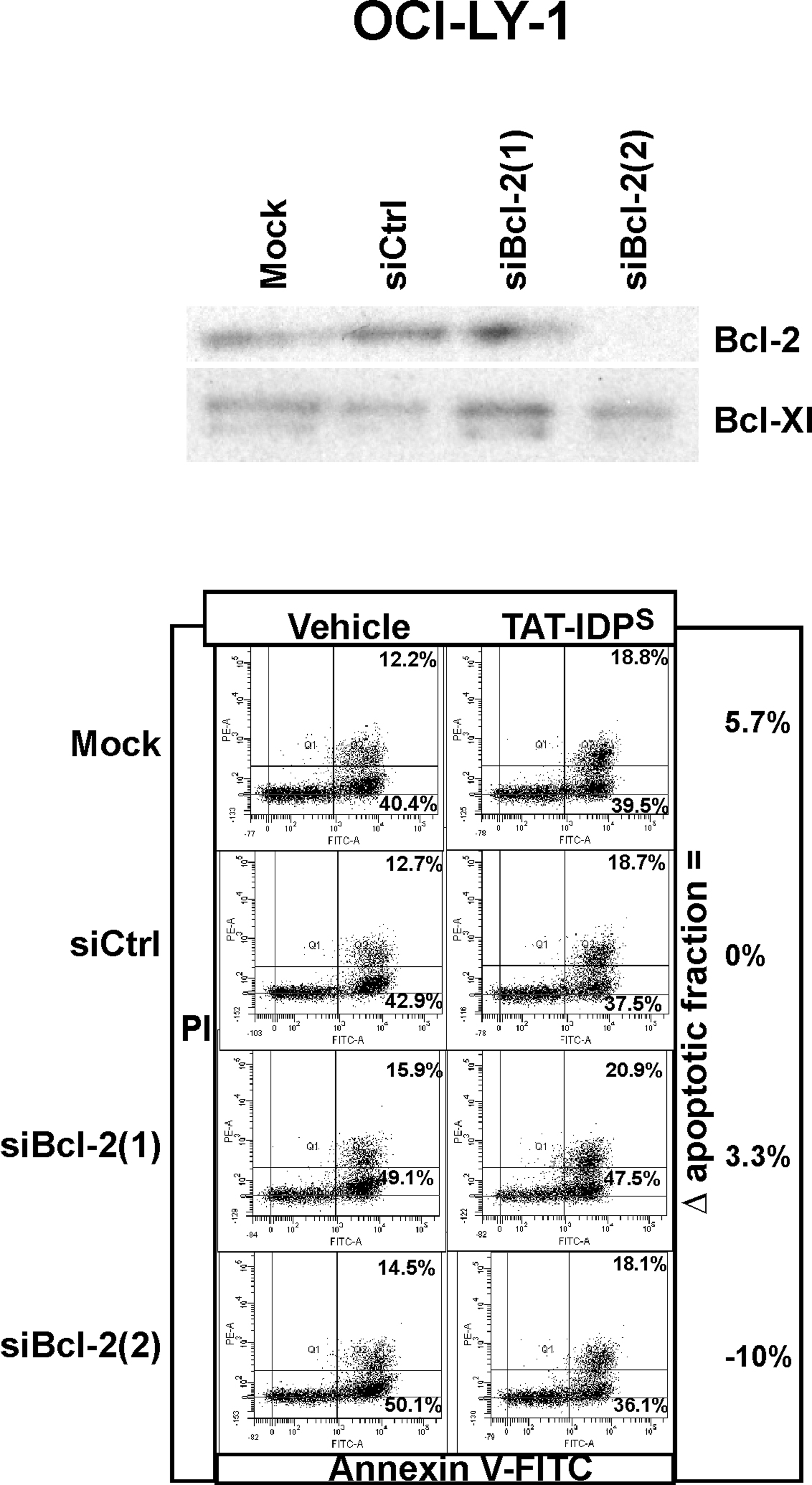

Supplement: Supplementary Figure S3 [file cddis2013140x4.tif]
